# Experimental and Numerical Investigation of the Extrusion and Deposition Process of a Poly(lactic Acid) Strand with Fused Deposition Modeling

**DOI:** 10.3390/polym12122885

**Published:** 2020-12-01

**Authors:** Anne Gosset, David Barreiro-Villaverde, Juan Carlos Becerra Permuy, Marcos Lema, Ana Ares-Pernas, María José Abad López

**Affiliations:** 1 Technological Research Center (CIT), Universidade da Coruña, Campus de Esteiro, 15403 Ferrol, Spain; juan.becerra@udc.es (J.C.B.P.); ana.ares@udc.es (A.A.-P.); maria.jose.abad@udc.es (M.J.A.L.); 2 CITIC Research, Universidade da Coruña, Campus de Elviña, 15071 A Coruña, Spain; david.barreiro1@udc.es (D.B.-V.); marcos.lema@udc.es (M.L.)

**Keywords:** thermoplastic, high-speed visualization, computational fluid dynamics, single strand deposition, high printing speeds

## Abstract

In the last decade, Fused Deposition Modeling (FDM) has gained popularity for allowing the fabrication of pieces with complex shapes. The final quality of the pieces is strongly linked to the shape, size and surface finish of the strands deposited successively, which themselves depend on the printing parameters and extruded material properties. In this work, we present an experimental characterization of an extruded and deposited single strand of Poly-Lactic Acid (PLA), by means of high-speed visualization of the bead region between the substrate and the nozzle, where the molten polymer is still in liquid phase. A Computational Fluid Dynamics (CFD) model proposed in literature, and, based on isothermal and viscous flow assumptions, is validated with this data in terms of strand height and meniscus shape. The characteristics of the printed layer are also confronted to the measurements of the solidified strands by microscopy, with a good agreement. The focus on high printing speeds allows extending the conclusions of previous studies. Regarding the surface finish, the roughness patterns detected on the printed strands are correlated to the velocity fluctuations of the printing head. The CFD model does not capture those thickness variations, however, due to not accounting for solidification.

## 1. Introduction

Fused Deposition Modeling (FDM), also known as Fused Filament Fabrication (FFF), is an additive manufacturing (AM) technique that relies on the extrusion of a molten material through a moving nozzle. The strands thus deposited on top of each other conform the shape of a three-dimensional object. Although this technique was initially used for prototype making, the improvements reached during the last decade in terms of process control and material development have led FDM to become a major technique for the production of finished parts. Like any additive manufacturing technique, FDM has undeniable advantages regarding the fabrication of complex shaped pieces, minimizing the waste of material as no machining is required. It allows for the production of small series of pieces, with a remarkable flexibility for switching from one product to another.

**Figure 1:**
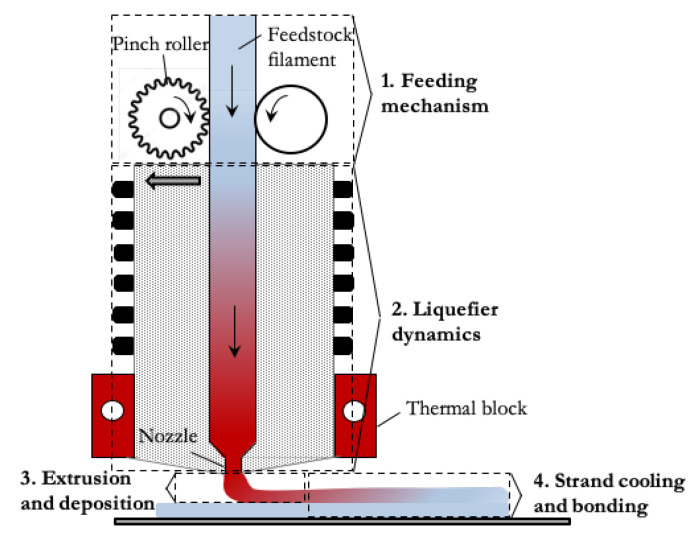
Sketch of 3D printer head and different sub-processes in Fused Deposition Modeling (FDM).

The most common materials used in FDM are thermoplastics. In desktop 3D printers, the feedstock is a filament which is forced into a liquefier, and its role is to melt the material at an adequate temperature for its extrusion through a small diameter round nozzle (a few tenth of millimeter). Because the latter induces a considerable head loss through the printer’s head, the filament is driven by pinch rollers which overcome the pressure loss with as little slippage as possible. Once extruded and laid down, the strands cool down and bond to the previously deposited layers. These different sub-processes are detailed in Reference [1], and represented in Figure 1.

The resulting structure in layers features a surface finish that is not as good as with other AM techniques, such as Stereolithography (SLA). Finished parts usually have a rough surface which is difficult to correct a posteriori, and suffer defects, such as warping [2] or delamination [3]. In addition, the strand shape and alignment affect the void between layers, as well as the quality of bonding when they cool down [4,5]. For an extensive review of the defects and treatments of FDM-based printed objects, the reader is referred to Reference [6]. It is thus crucial to control precisely the extrusion and deposition process of the molten polymer in order to obtain a final piece with optimum properties.

Up to now, a great deal of effort has been dedicated to the determination of optimum process windows for FDM [7]. The procedures for that range from statistical design optimization [8,9], to Grey Relational Analysis (GRA) [10], or artificial intelligence techniques [11,12], in order to identify the combination of parameters that influence the characteristics of the final piece. However, these procedures were developed for one single material at a time, usually a common thermoplastic, such as Poly-Lactic Acid (PLA) or Acrylonitrile Butadiene Styrene (ABS), and the results can hardly be extrapolated to a new material [7,10]. The lack of a generalized procedure to optimize printing parameters makes numerical modeling a good candidate to predict the thermal and fluidic behavior of a given polymer through the printer’s head [12]. In addition, it eliminates the need for a large number of experiments.

The different modeling approaches potentially applicable to FDM are presented in the very complete review of Bikas et al. [13]. The simulation of the full process, from the forcing of the solid filament into the liquefier, to the extrusion of the molten material and its posterior cooling and bonding to adjacent strands (Figure 1), is very complex because it involves a wide range of physical phenomena (mechanisms, heat transfer, phase change, non-newtonian and viscoelastic behavior of the melt, molecular diffusion during bonding, etc.). As suggested in Reference [1,14], the different sub-processes can be simulated in an independent manner. Taking care that the simulations of the different steps are thoroughly validated, the output of one sub-process may be the input of the next one.

For example, the melt flow in the liquefier was computed through Finite Volume Methods in Reference [15,16], among the most recent contributions. The extrusion and deposition of the melt, which is the sub-process we are interested in here, was simulated using Finite Element methods [17,18]. Based on the numerical results, Agassant et al. [18] even derived an analytical model that relates the dimensions of the strand, and other variables, such as the pressure at the extrusion nozzle, to the printing parameters. Hebda et al. [19] also proposed a simple model to predict the strand size, based on a simple argument of mass conservation between the nozzle exit and the deposited layer, and an empirical correlation for the solidified strand width. However, most of the latest works use Finite Volume Methods for the computation of the highly viscous polymer flow. These studies differ from each other by the assumptions made: Du et al. [20] carried out two-phase simulations, assuming a purely viscous non-newtonian behavior of the melt, and accounting for heat transfer as the process is laser-assisted. Xia et al. [21] proposed fully resolved simulations, including heat transfer and shear thinning behavior, and even studied the solidification and residual stresses in a companion paper [22]. Comminal et al. [23] considered an isothermal and newtonian flow, arguing that in the creeping flow regime, the predictions are little affected by the local viscosity. Since the latter is mostly dependent on the shear rate and temperature, heat transfer and shear thinning behavior are disregarded. This simplified approach seems rather convincing since a good qualitative agreement is found between the predicted strand cross sections and the morphology of the actual solidified strands [24], within the range of printing parameters investigated. Behdani et al. [25] noticed, however, that the agreement is getting worse at small nozzle to substrate separation, and they propose a non-isothermal, no-newtonian fluid model with which they reach a better correspondence between predictions and the experiments in Reference [24]. Most of these last studies contemplate the deposition of one single strand on a flat substrate, but it is worth mentioning that the building of more complex shapes has recently been considered in Reference [26,27].

An important question is related to the importance of viscoelastic effects in FDM. As well known in extrusion, those effects materialize with the formation of a die swell at the nozzle exit. Comminal et al. [28] showed that the shear thinning behavior reduces the extrudate swell in the simple extrusion of a purely viscous power law fluid and a viscoelastic Giesekus fluid. However, the strong interaction between the extruded flow and the solid substrate in FDM makes it difficult to extrapolate those results and evaluate the importance of viscoelastic effects without simulating the actual flow.Xia et al. in Reference [29] found that, during extrusion and deposition in FDM, the inclusion of viscoelastic effects increases the size of the bead, with the evidence of a die swell forming right at the slot exit. It leads to a slightly larger layer thickness after deposition, compared to a purely viscous flow. This effect is attenuated when the strand is topped by another filament. It should be underlined that in this study, a very simplified nozzle is used, with no rims; it can be expected that the presence of flat rims, like usually found in printing nozzles, prevents the free development of the extrudate swell, and reduces its influence on the bead. Liu et al. [30] presented a CFD study in which viscoelasticity and solidification are accounted for, this time with a more realistic geometry of the nozzle. However, the computations are 2D, and no comparison with fully viscous computations, nor experimental validation, were provided to quantify the importance of die swelling.

Finally, there is a topic which has not been tackled in literature: the wetting behavior of the molten polymer on the substrate. It is known to be poor in the case of thermoplastics, which is why spray adhesives or other solutions, like Kapton tape, are frequently used to ensure the adhesion of the first layer on the hot bed. The wetting characteristics of a liquid on a given surface are determined by the contact angle between the liquid interface and the substrate at the triple line, where the liquid, gas and solid meet. They are closely linked to the liquid surface tension, as well as the interfacial tension between the solid and the liquid. It is of interest here, since our study focuses on the bead deposited in liquid phase and the resulting meniscus. In fact, the extrusion and deposition process in FDM is very similar to the slot die coating process, widely used in the paper and battery electrodes industry [31]. While this process is normally bidimensional, with a long slot die delivering the coating on a flat substrate, FDM can be seen as the axisymmetric version, with a round die. The stability and defect free window of slot die coating is strongly influenced by the behavior of the dynamic contact angles formed by the meniscus [31], and the stability of the bead is frequently ensured by applying vacuum on the upstream side of the die. Molten thermoplastics, such as the ones used in FDM, usually exhibit large equilibrium contact angles (>90°) on non-treated substrates, such as polished metals and glass [32,33]. Besides, this angle is known to increase with the zero shear viscosity of the material [34], that is to say, when the melt temperature decreases. The temperature difference between the extruded melt and the heated platform, which is very large in 3D printers, is also expected to affect the wetting behavior, as opposed to slot die coating. The effect of the surface temperature on the dynamic contact angle is studied in sessile drop spreading experiments with a thermoresponsive polymer in Reference [35] and alkanes in Reference [36]. In both cases, it is found that an earlier contact line arrest (due to solidification in Reference [36] or to gelation in Reference [35]) leads to larger contact angles compared to isothermal configurations. The study by Pan [37] is the only one led in conditions close to the actual ones in 3D printers: a polyethylene strand is deposited on aluminium and Polyethylene Terephthalate (PET) substrates at different temperatures. The analysis of the post-solidification strand shape indicates that the lateral contact angles tend to increase as the surface temperature is decreased, in agreement with the previous references. No measurements are reported for PLA or other common thermoplastics used in FDM.

As for capillary effects, Crockett [38] states that the spreading of beads is mostly driven by surface tension in the extrusion of ceramic slurries. However, the molten polymers used in desktop 3D printers have vastly different properties. In Reference [20,21,26], surface tension effects are included in the two-phase computations, but the contact line treatment is not specified. In Reference [23], the authors disregard this effect as they consider a creeping flow regime in which viscosity dominates all other forces.

As a conclusion, several numerical approaches have been developed to simulate the extrusion and deposition process in FDM, with a varying degree of approximation. However, very few have been validated with experimental data, due to their scarcity. Besides, to our knowledge, there has been no attempt to examine the quality of the predictions in the bead, i.e., the small region of interaction between the extruded flow and the platform, where the polymer is still liquid. It is an important region, since the behavior of the meniscus formed here determines the limits of the process in terms of printing speed.

In this work, the extrusion and deposition of a single strand of PLA is characterized by means of high-speed visualization of the bead region between the substrate and the nozzle. The data is used to validate CFD simulations of the process, adopting a simplified approach in which the flow is assumed fully viscous and isothermal. An emphasis is put on the prediction of the meniscus shape. The experimental animations allow for an accurate determination of the printing parameters used as inputs in the computations, particularly the printing speed. The strand characteristics while in liquid phase are also correlated with the cross section and surface finish of the final solidified strands. We focus on large print to extrusion velocity ratios, that is, on fast printing conditions, with small strands.

## 2. Materials and Methods

### 2.1. Experimental Measurements

#### 2.1.1. Setup

**Figure 2:**
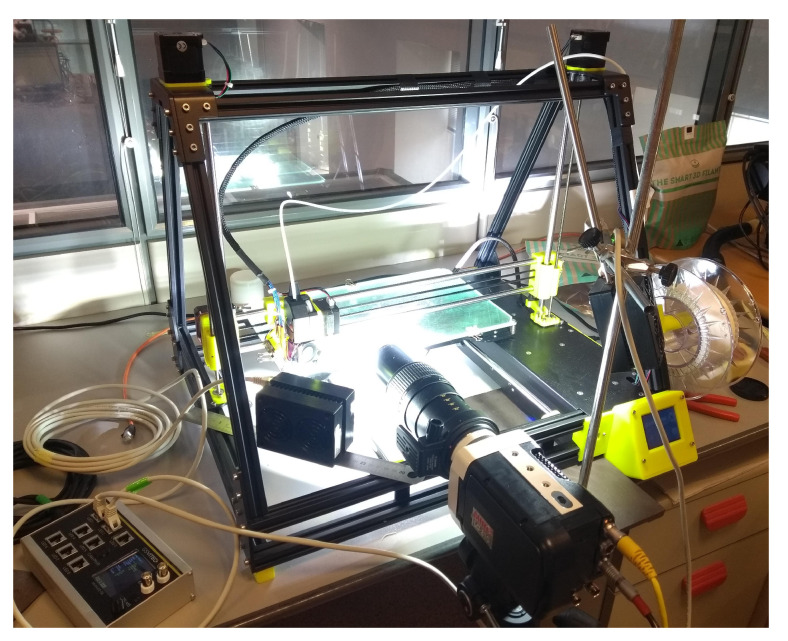
Experimental setup with high-speed camera and illumination.

The experiments were conducted on a desktop open-source MendelMax XL V6 3D printer (MakerGal, Santiago de Compostela, Spain). A simple perimeter was printed, for which the G-code was manually written. The high-speed visualizations were carried out during the printing of the first 278-mm-long straight line. A Phantom camera was used, with a Canon macro-lens to capture correctly the meniscus region. The optical axis was carefully adjusted to be perpendicular to the printing head plane. A frame rate of 3600 FPS, with an exposure time of 140 μs, was deemed sufficient to capture accurately the motion of the printing head and the deposited bead. The time resolution of the animations is, thus, of 0.28 ms. The region was illuminated by an array of pulsed LEDs synchronized with the camera acquisition, in which light was diffused with translucent paper (Figure 2). The spatial calibration was carried out with a single shot of a calibrated shim 500±1 μm thick, laying down on the heated bed. The resulting spatial resolution of the images is 9.4 μm/pixel ±2%, leading to a spatial accuracy of ±19 μm. The experiments were carried out with commercial PLA (Smartfil, ivory) at a temperature of 215±1 °C, while the substrate was maintained at 60±1 °C. The filament had a diameter $D_{f}=1.75\pm 0.05$ mm.

Prior to the experiments, the leveling of the bed was adjusted and the nozzle to substrate separation *h* was checked by using shims of different thickness. Then, the rate at which the filament enters the liquefier was calibrated against the roller motor steps per millimeter established by the constructor as a function of the filament and nozzle die diameters, respectively, $D_{f}$ and *d*. For that, different filament lengths were marked and the filament was advanced manually into the printing head to determine the actual steps/mm of filament. The calibration was finally checked with the printing of a cube of lateral wall thickness 0.3 mm, with a nozzle of diameter d=0.4 mm and a layer width of 0.3 mm. All the experiments were carried out with an Olsson Ruby nozzle of 0.4 mm.

Two values were considered for the nozzle to substrate separation *h*, or layer thickness: 0.2 and 0.3 mm, corresponding to normalized separations h/d=0.5 and 0.75. For each of them, three different print speeds were considered (15, 22, and 30 mm/s), with the extrusion volumetric rate adjusted to reach a strand width of approximately 0.4 mm. This means that the ratio of the printing speed *V* to the mean extrusion speed *U* is kept constant while varying *V*. This ratio V/U is equal to 2 for h/d=0.5 and 1.3 for h/d=0.75. The test conditions are detailed in Table 1. Note that the printing and mean extrusion velocities correspond to the target values in the G-code, and the actual value of these parameters is estimated during the experiments.

Before each experiment, the nozzle was purged. Most of the tests were repeated three times, with a repetability that is illustrated in Section 3.

For the calculation of the mean extrusion velocity in the computations, the length of filament that enters during the straight line printing was controlled by the G-code: it was equal to 11.0 mm for h/d=0.75 and 7.3 mm for h/d=0.5. By virtue of volume conservation, the volumetric flux ${\dot{Ɐ}}_{f}$ of solid filament that penetrates into the liquefier should be equal to the extruded flux of material ${\dot{Ɐ}}_{ext}$, corrected for thermal expansion:

(1) $${\dot{Ɐ}}_{ext}=\left(1+3\alpha_{L}\Delta T\right){\dot{Ɐ}}_{f}\Rightarrow \pi \frac{d^{2}}{4}U=\left(1+3\alpha_{L}\Delta T\right)\frac{D_{f}^{2}}{4}U_{f},$$

where $U_{f}$ is the velocity of the filament, $\alpha_{L}$ is the coefficient of linear thermal expansion of PLA, and ΔT is the temperature difference between the ambient and the molten PLA (215 °C). In this approximation, it is assumed that the volume variation due to thermal expansion in the liquefier remains small.

However, because of multiple sources of error on $U_{f}$ (slippage of the filament in the gear system, among others), a calibration coefficient is to be applied to Equation (1) [24]. For that, the real flux of deposited material ${\dot{Ɐ}}_{s}$ is measured from the cross-sectional area of the solidified deposited strand, $A_{s}$, and it is assumed equal to the extruded flux (with thermal correction):

(2) $${\dot{Ɐ}}_{ext}=\left(1+3\alpha_{L}\Delta T\right){\dot{Ɐ}}_{s}\Rightarrow \pi \frac{d^{2}}{4}U=\left(1+3\alpha_{L}\Delta T\right)A_{s}V.$$

Using Equations (1) and (2), with *V* the printing head velocity measured from the high-speed images, we find that the ratio of the input filament flux to the output strand flux lies within a range from 1 to 1.1, with an average value of 1.03. As in Reference [24], the ratio ${\dot{Ɐ}}_{f}/{\dot{Ɐ}}_{s}$ is larger than unity, which is somehow surprising as we expected the main reason for the deviation to be the slippage of filament. However, we do not find values as high as 1.30, as in this last reference. The exact estimation of the printing speed is probably the reason why the ratios are so close to one. The exact value for each test is used in the computations.

#### 2.1.2. Rheological Characterization of Material

The rheological characterization of PLA was performed at the temperature of 215 ± 1 °C using a controlled strain rheometer (ARES, TA Instruments, New Castle, DE, USA ) with parallel-plate geometry (25 mm diameter, 1 mm gap) under nitrogen atmosphere. The steady shear viscosity, η, was measured in a range of shear rates between 0.1 and 1000 s^−1^.

**Figure 3:**
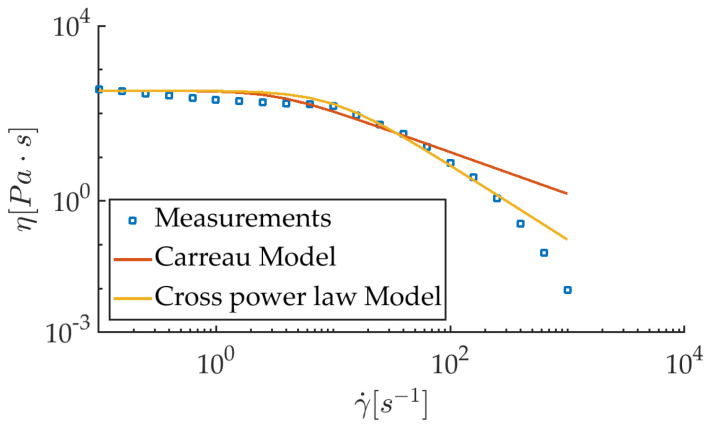
Dynamic apparent viscosity of the poly(lactic acid) (PLA) used for experiments.

As expected with this kind of neat polymer, PLA displays a shear thinning behavior after the newtonian plateau (Figure 3). The viscosity curve is better fitted with a cross power law model (used in Reference [21], for example) than with a classical Carreau-Yasuda model, as in Reference [39]. The steady viscosity features the following dependence on the shear rate $\dot{\gamma }$:

(3) $$\frac{\eta -\eta_{\infty }}{\eta_{0}-\eta_{\infty }}=\frac{1}{1+{\left(K\cdot \dot{\gamma }\right)}^{n}},$$

where $\eta_{0}$ is the zero shear rate viscosity, $\eta_{\infty }$ is the infinite shear rate viscosity (taken equal to zero here), *K* is the natural time (inverse of the shear rate ${\dot{\gamma }}^{*}$ at which the fluid changes from newtonian to shear thinning behavior), and *n* is the power law index. With the time constant fixed at $K=1/{\dot{\gamma }}^{*}=0.1$, least square fitting of the data points leads to n=1.7, with $\eta_{0}=330$ Pa·s. This model is the one introduced in the numerical computations that account for the non-newtonian behavior of PLA.

#### 2.1.3. Image Processing

**Figure 4:**
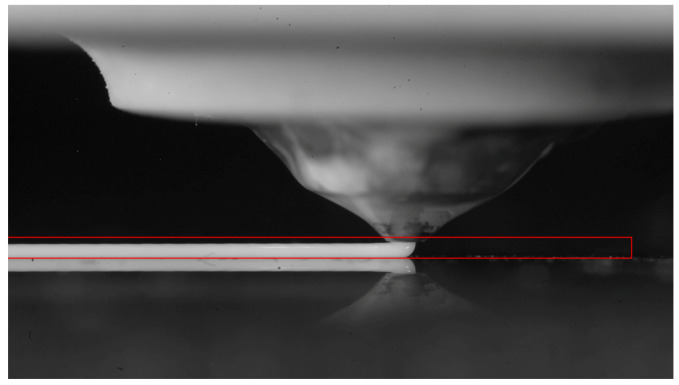
Full resolution snapshot of a high-speed video.

**Figure 5:**
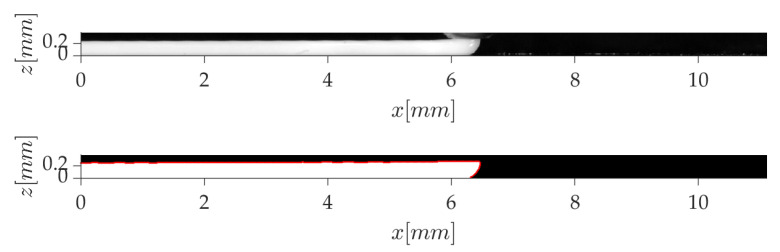
Detection of strand contours for the estimation of printing speed.

The high-speed animations were processed with MATLAB to extract the strand contours, as well as the instantaneous velocity of the printing head. The strand region was first cropped from the original snapshot (Figure 4). After contrast adjustment, the image was binarized, and small white pixel regions due to defects were eliminated with morphological operations. The external strand contours were finally detected, as in Figure 5.

**Figure 6:**
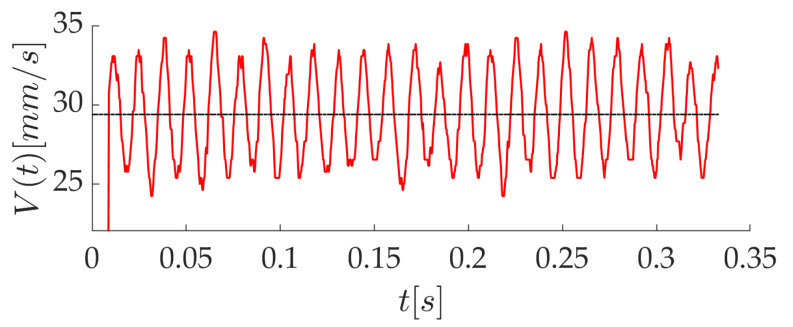
Time evolution of the printing head speed.

The instantaneous velocity of the printing head is computed from the position of the strand front at two time instants separated by 5 to 8 ms (depending on *V*), which corresponds to a displacement between 125 and 167 ±9μm. At every time step, a running average is performed over the two last velocities. The time evolution of *V* is quasi sinusoidal, as in Figure 6, for a mean velocity $\bar{V}=29.4$ mm/s. The mean amplitude of the oscillations can be approximated by the standard deviation of the signal, and it is equal to 2.8 mm/s in the present case. This behavior is found for all printing speeds, with variations in the frequency and amplitude of the fluctuations. It is due to the use of a tooth belt for the motion of the printing head.

#### 2.1.4. Characterization of the Deposited Strands

After the tests, the morphology of the solidified strands was analyzed using a using a JEOL JSM-7200F Field Emission Scanning Electron Microscope (Tokyo, Japan), with a magnification ×200. The samples were first cut at ambient temperature with a razor blade, but the deformation of the strand turned out to be visible. Therefore, they were introduced in liquid nitrogen and cryogenically broken to avoid the plastic deformation on fracture surfaces and strand section. Longitudinal views of the samples were also taken, in order to have an accurate measurement of the road width and to detect the deformations of the strand.

### 2.2. Numerical Simulations

#### 2.2.1. Numerical Models and Approximations

The numerical simulations were carried out with the two-phase incompressible flow solver *interFoam* of the open-source finite volume libraries *OpenFOAM*. The tracking of the interface between the molten polymer and the air is based on the Volume of Fluid (VOF) method. The later relies on the transport of a color function which represents the liquid volume fraction β in each computational cell. In a given control volume, β is equal to 1 when it is filled with liquid, and 0 when it is filled with gas. The interface is assumed to be located in the cells where 0<β<1. The local fluid properties ϕ (density and viscosity) are computed as a weighted average of the properties of the liquid $\phi_{l}$ and gas $\phi_{g}$, depending on β:

(4) $$\phi =\phi_{l}\beta +\phi_{g}\left(1-\beta \right).$$

The fluid properties are introduced in the incompressible Navier–Stokes Equations (5) and (6) that govern the viscous flow, together with the transport Equation (7) for the volume fraction β.

(5) ∇·u=0,

(6) $$\rho \left(\frac{\partial u}{\partial t}+u\cdot \nabla u\right)=-\nabla p+\rho g+\nabla \cdot \left[\eta \left(\nabla u+\nabla^{T}u\right)\right]+S_{\sigma },$$

(7) $$\frac{\partial \beta }{\partial t}+u\cdot \nabla \beta =0,$$

where u is the velocity vector, *p* the pressure, ρ and η the fluid density and dynamic viscosity, respectively, g the acceleration of gravity, and $S_{\sigma }$ the Laplace pressure term that accounts for surface tension effects.

The *interFoam* solver used here relies on an algebraic VOF method and does not include a geometrical reconstruction of the interface. Instead, an artificial relative velocity term is activated in order to compress the gas-liquid boundary and maintain the interface as sharp as possible. The interface is recovered as the isosurface corresponding to β=0.5.

The flow is considered isothermal in the computations, as in Reference [23]. As we are mostly interested in the region right after extrusion, the variation of temperature, and thus of viscosity, is assumed to be small enough to have no influence in this inertialess flow regime. Both newtonian and shear-thinning viscous flow behaviors are considered in the forthcoming computations. Viscoelastic effects are disregarded. The computational time step is adjustable and usually around $10^{-5}$ s.

#### 2.2.2. Flow Conditions and Domain

**Figure 7:**
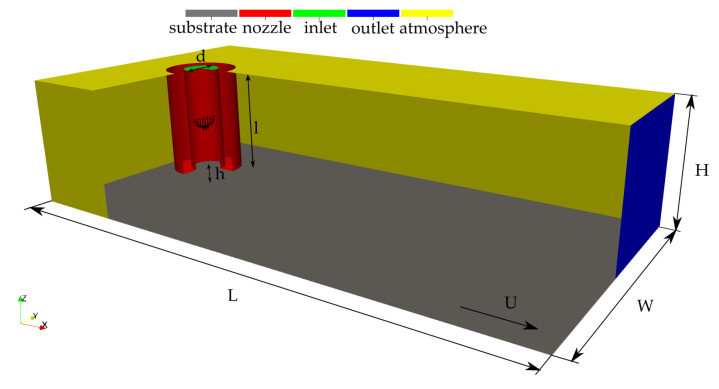
Computational domain and boundary conditions.

The simulations reproduce the exact conditions of the experiments described in Section 2.1, with the measured printing velocity and the estimated extrusion velocity. The domain, represented in Figure 7, includes the final part of the extrudor nozzle, which is 1.3 mm long, with 0.2 mm thick rims. It extends over a distance L=6 mm in the direction *x* of motion. Its total width is W=3 mm and its height, *H*, varies between 13.2 and 13.3 mm. The relative motion between the printing head and the substrate is simply ensured by imposing a velocity to the bottom wall, instead of moving the printing nozzle. The boundary conditions include no-slip for the substrate and the nozzle walls, and typical outlet conditions for the surrounding patches in the ambient. At the inlet of the nozzle, a Hagen-Poiseuille developed velocity profile is imposed:

(8) $$u\left(r\right)=2U\left(1-\frac{r^{2}}{{\left(d/2\right)}^{2}}\right)0\leq r\leq d/2,$$

with *u* the velocity component in the vertical *z* direction, *r* the radius, and *U* the average extrusion velocity.

The presence of a contact line at the intersection of the polymer, the substrate, and the air makes necessary the definition of a boundary condition for β on the walls. A Neumann condition is applied in most of the calculations, even if some trials of imposing a dynamic contact angle as a function of the contact line velocity were also carried out.

The material properties are detailed in Table 2.

**Figure 8:**
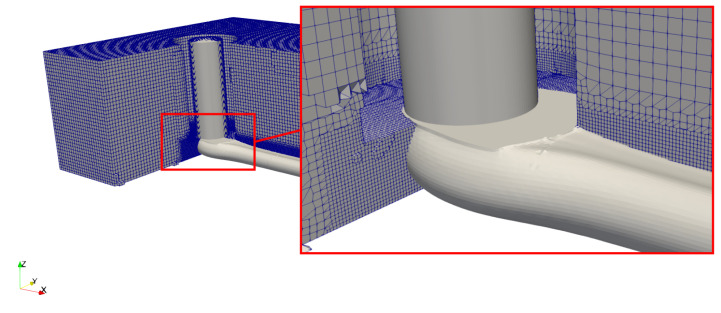
Computational grid.

The computational grid was designed using the opensource libraries *cfMesh*. It is based on a cartesian mesh which is successively refined in the regions of interest and towards the boundary surfaces. It contains mostly hexahedral cells, except in the transition regions from one level of refinement to the next. Three different meshes were used for the grid independency study, for which characteristics are given in Table 3. The base mesh represented in Figure 8 features cubic cells of size 80 μm, refined to 20 μm in the region of the strand.

The characteristic flow Reynolds number Re=ρUd/η, which represents the ratio of inertia to viscosity forces, ranges between $10^{-5}$ and $3\times 10^{-5}$. This indicates a creeping flow regime dominated by viscosity.

## 3. Results and Discussion

### 3.1. Numerical Results-Sensitivity to Modeling Parameters

**Figure 9:**
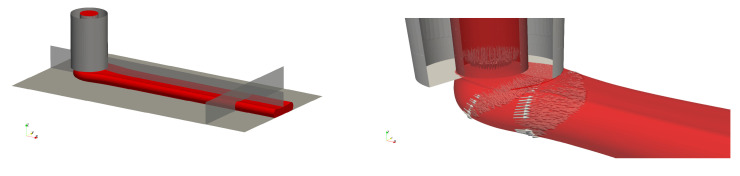
Example of strand shape for h/d=0.5 and V/U=1 with the transversal and longitudinal cutting planes. Close-up of the velocity distributions near the nozzle exit.

A typical strand shape obtained in the two-phase simulations is presented in Figure 9 for h/d=0.5 and V/U=1. It can be seen how the nozzle rims flatten the strand and force it to spread laterally. There is a full contact between the extruded material and the nozzle edges, so that the strand height is essentially dictated by the nozzle gap in these conditions. A liquid build-up also takes place on the meniscus side.

**Figure 10:**
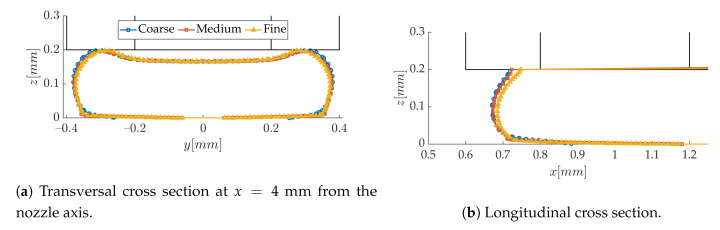
Mesh independency study based on the strand shape (*h/d* = 0.5, *V/U* = 1).

**Figure 11:**
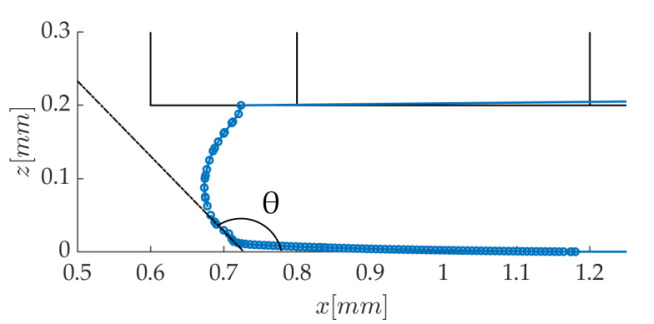
Dynamic contact angle of the meniscus.

Before confronting the numerical predictions to the experimental data, the sensitivity of the results to model parameters is checked. The flow behavior is first considered as newtonian, and surface tension is activated. It can be appreciated in Figure 10 that the medium mesh with 20 μm cells is capturing the strand cross-section as accurately as the fine one; it will therefore be used as the base mesh for this study. In this last figure, it is interesting to note that the contact angle, θ, between the liquid interface and the substrate (Figure 11) is very large. θ is determined from the tangent to the interface at a distance of about 10–15 μm from the substrate, as illustrated in Figure 11 in the meniscus. The resulting value thus corresponds to the apparent contact angle measured experimentally, at a rather “large” distance from the surface. The lateral contact angles on each side of the strand reach 134° and are expected to be close to the equilibrium value since the spreading process here is very slow. The dynamic contact angle in the meniscus is a receding one, which explains why it is lower (104°). In any case, these values are coherent with the contact angles measured in drop spreading experiments with molten polymers [34,40]. The contact line boundary condition was modified to impose different values of this contact angle, but the meniscus shape remained exactly the same (not shown here). This suggests that, at our observation scale, the near wall shape of the polymer flow is mostly governed by the viscous drag induced by the moving substrate, and surface tension plays an insignificant role.

**Figure 12:**
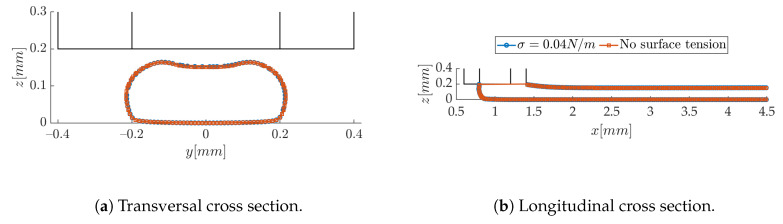
Effect of surface tension of the strand shape (*h/d* = 0.5, *V/U* = 2).

This is further checked by desactivating the surface tension term in Equation (6). It is obvious from Figure 12 that its effect is negligible, as suspected in Reference [26]. The characteristic flow capillary number Ca=ηV/σ, which measures the relative importance between viscous and capillary forces, ranges in our experiments from 60 to 200. This order of magnitude confirms that surface tension is irrelevant.

**Figure 13:**
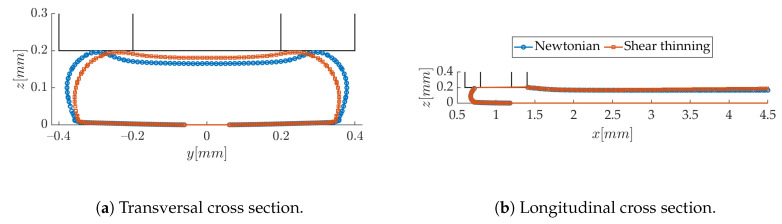
Effect of non-newtonian behavior on the strand shape (*h/d* = 0.5, *V/U* = 1).

Finally, the influence of the shear-rate dependent viscosity of PLA on the extrusion flow is checked. The cross-power law constitutive model (3) found from the rheological characterization is used in the *interFoam* solver, while, in the case of newtonian flow, the zero shear dynamic viscosity $\eta_{0}=330$ Pa·s is considered. In Figure 13, the comparison of the longitudinal and transversal profiles of the strand show that close to the extrusion region, the meniscus shape is weakly affected. After deposition, the strand is slightly less flat and wide when shear-thinning is accounted for. This is due to lower shear rates and, therefore, higher effective viscosity in this region. The same tendency was observed in Reference [41]. However, solidification has probably already taken place at x=4 mm from the nozzle axis, where the cross-section is taken; therefore, shear thinning effects are little relevant in our study.

**Figure 14:**
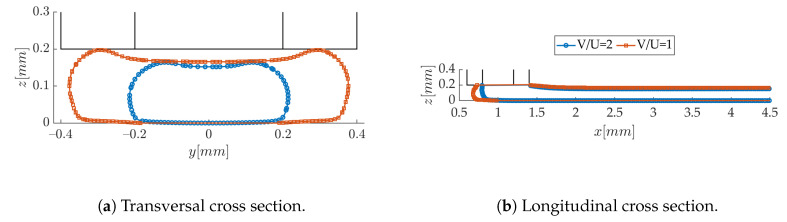
Effect of the velocity ratio on the strand shape at *h/d* = 0.5.

The effect of the printing to extrusion velocity ratio on the strand shape is illustrated in Figure 14a. As expected, the deposited polymer road is wider at smaller V/U values, for a constant separation *h*: this is because the extruded volumetric flux is larger compared to the printing speed. It can be seen in Figure 14b that the meniscus features a more prominent bulge, due to the spreading of the bead. It is also interesting to note that the layer height is smaller than *h* for V/U=2, while it is equal to *h* for a ratio of 1. In the latter case, one can observe how the contact between the nozzle rims and the strand sets the layer height, even if there is a slight depression in the center (also observed in Reference [23] in similar conditions). At the larger printing speed V/U=2, the interaction between the deposited stream of melt and the nozzle is weaker (at least on the bead side); therefore, the strand is more “free” to set its height. Comminal et al. [23] state that the common approximation that the layer height *H* equals the nozzle separation *h* is valid only when $h<d\sqrt{U/V}$. While it is clearly verified for V/U=1, this relation does not hold for the highest ratio. However, the authors in Reference [23] did not investigate such a high velocity ratio range. This finding might be of interest for further studies focusing on high printing speeds.

**Figure 15:**
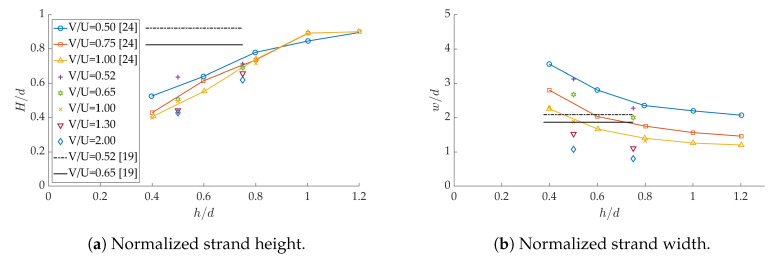
Comparison of strand dimensions with previous studies.

For different values of V/U between 0.5 and 2, the normalized strand height H/d and width w/d are confronted to the results of [19,24] in Figure 15. At V/U=1, both dimensions are in good agreement with Reference [24]. The height is consistently lower than *h* for the higher ratios V/U=1.3 and 2. As for the width, the decreasing trend of *w* as the velocity ratio is increased is confirmed. It is interesting to note that the simple model of Hebda et al. [19] predicts constant strand dimensions when h/d is varied over an interval [0.37–0.75], with V/U in a similar range than ours. In addition, the layer height is overestimated, and the width underestimated, especially at small nozzle gaps. This is clearly in contradiction with the results in Reference [24] and the present study, in spite of the very similar printing conditions. The reason behind this trend is that Hebda’s model is based on an empirical correlation between *w* and V/U which dismisses the effect of h/d; the latter is weak, but present (see Figure 7 in Reference [19]). Therefore, care should be taken when using this type of simplified model.

### 3.2. Confrontation of Numerical and Experimental Results

**Figure 16:**
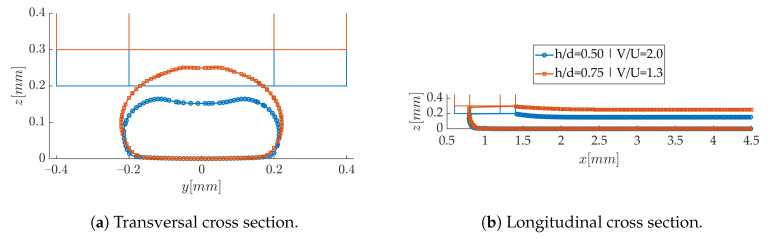
Effect of nozzle separation on the strand shape.

The experiments have been carried for two nozzle separations, with a velocity ratio adjusted so as to give the same layer width. The printing speed was varied, keeping the velocity ratio constant. This joint effect of h/d and V/U is illustrated in Figure 16. As expected, the layer width is almost unaffected by the modification of printing parameters (Figure 16a), since the velocity ratio is adapted for that purpose. The shape of the meniscus is very similar in both cases (Figure 16b), emphasizing its correlation with the strand width. The V/U ratio being larger than unity in both cases, it is not surprising to find that the resulting layer thickness layer is smaller than *h*: the deposited volumetric flux is small in relation with the printing speed. It points out that the usual rule in 3D printers “layer thickness = nozzle separation” does not hold for high printing speeds. Finally, when V/U is maintained constant as the printing velocity is modified, the strand shape remains the same in the computations, since it is entirely determined by the extruded volumetric flux and volume conservation. However, in the experiments, the measurement of the printing head speed and the strands cross-sectional areas show that this ratio is not exactly constant. The velocities have thus been adjusted in the simulations for their validation.

**Figure 17:**
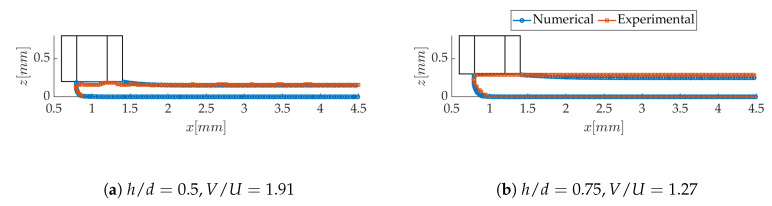
Comparison of numerical and experimental longitudinal strand shapes in liquid phase. The experimental contours are derived from the procedure described in Section 2.1.1.

The longitudinal profiles of the strand are first confronted to the high-speed images, when the melt flow is still in liquid phase. Two examples are shown in Figure 17, for the two nozzle separations. The shape of the meniscus is, in general, very well recovered, within the spatial resolution of the images. For the largest value of *h*, however, the strand height remains constant in the experiments, while it decreases slowly in the computations. In this downstream region, crystallization and solidification have probably stopped the spreading of the layer, so this trend is not considered significant.

When extracted from the numerically predicted strand shapes in Figure 17, the dynamic contact angle of the meniscus is estimated at 140° for h/d=0.5 and 144° for h/d=0.75. In the experimental profiles of the same figure, it varies in a range of 130± 10° for h/d=0.5 and 145± 14° for h/d=0.75. The increasing trend of θ with h/d is therefore captured by the computations. As for the lateral contact angles, they can only be compared to the ones measured on the solidified samples. Both the experimental and numerical values of θ are found to increase with h/d, as does the meniscus contact angle. The modification of the strand cross section from a flat ovoid to a rounder shape when h/d increases explains this variation. The contact angle on the final strands rises from $110\;\pm \;9^{{}^{\circ}}$ to 122 ± 10° when h/d increases from 0.5 to 0.75, while it rises from 112 to 118° in the computations. The values are surprisingly close (although subject to a high uncertainty), considering the process of contact line arrest by solidification is not accounted for in the simulations.

**Figure 18:**
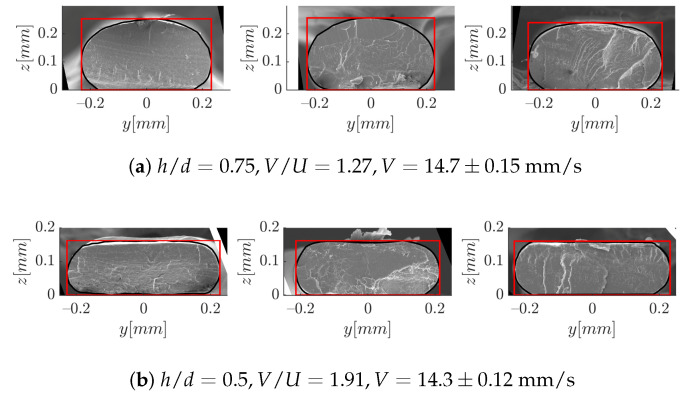
Illustration of the repetability of strand deposition.

In spite of their interest to analyze the meniscus region, the high-speed animations do not provide information on the layer width. For that, the solidified strands were used, as in Reference [24]. The repetability of the measurements is illustrated in Figure 18, for the two values of h/d. Each image corresponds to an independent experiment.

**Figure 19:**
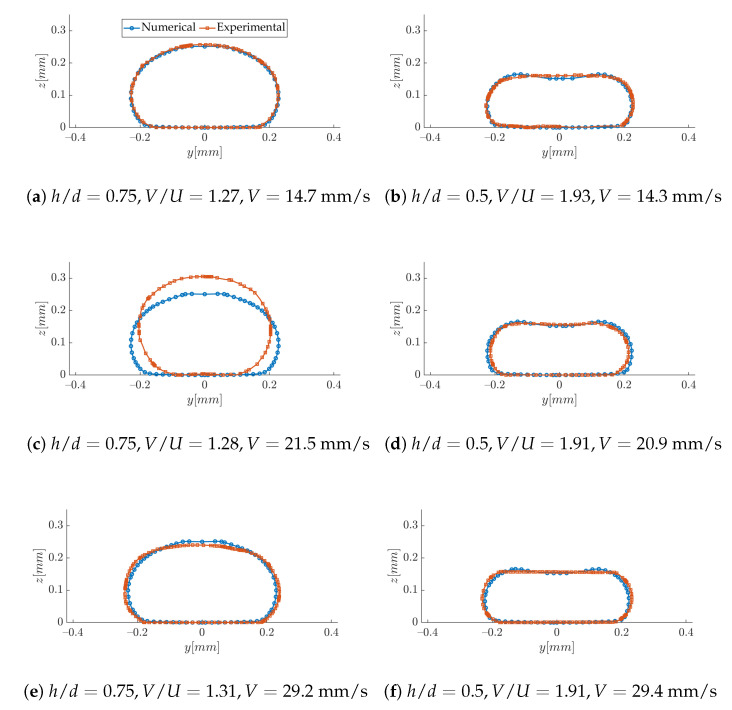
Comparison of strand cross-section.

The comparison of the strand shape in Figure 19 is done independently for every case, for which the exact process parameters were introduced in the numerical model. The experimental shape results from the averaging of several samples (3 to 5, according to the quality of the images). The agreement is in general very good, except for the case h/d=0.75 with the intermediate printing speed. We suspect this is because of problems we experienced when the samples were cut in liquid nitrogen. In spite of this particular case, the results indicate that, even when viscoelastic and thermal effects are disregarded, the CFD model used here is a reliable tool to predict the final shape of the printed filaments, within the range of printing parameters under study.

### 3.3. Influence of the Printing Head Velocity Fluctuations

**Figure 20:**
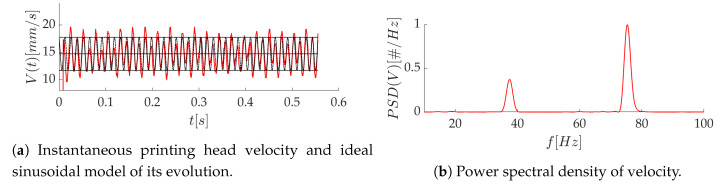
Time evolution of the printing head velocity and frequency analysis.

**Figure 21:**
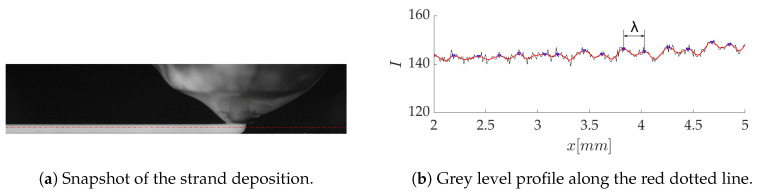
High-speed snapshot of the deposited strand for *h/d* = 0.75, *V/U* = 1.27 and *V* = 14.7 mm/s.

The important fluctuations of the printing head velocity led us to analyze their influence on the melt flow and the surface finish of the printed layer. For example, at a mean printing speed $\bar{V}=14.7$ mm/s, the time evolution of the velocity is nearly sinusoidal (Figure 20a), with a dominant frequency of F=75 Hz (Figure 20b) and a relative amplitude $A_{v}/\bar{V}=0.2$. For the same case, the observation of the high-speed images in Figure 21 reveals fluctuations of the grey level right below the interface, which reflects thickness variations in the deposited layer. The grey level profile in Figure 21b features an average fluctuation wavelength $\bar{\lambda }=0.213$ mm. This value can be related to the oscillating motion of the printing head, characterized by spatial fluctuations of wavelength V/F=0.196 mm. It seems, therefore, that the oscillations of the nozzle are “printed” on the deposited layer and participate to the overall roughness of the strands. Other types of instabilities typical in extrusion processes, such as the sharkskin defect [42], have been evoked to explain the roughness of the strand [15], but the strong correlation found here suggests that it is mostly due to the print head motion in the desktop 3D printer used in this study.

**Figure 22:**
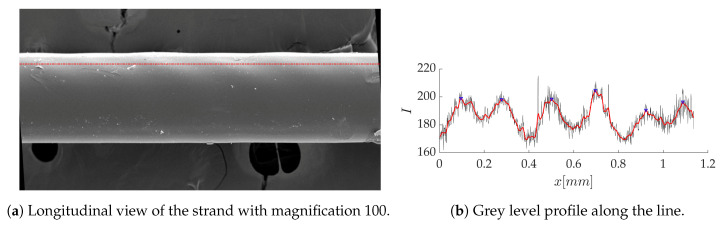
Longitudinal analysis of the solidified strand.

The magnified top view of the corresponding sample shows that the thickness fluctuations remain on the strand after solidification: we can observe, in Figure 22a, an oblique regular pattern, with an average wavelength of 0.198 mm (Figure 22b) that is coherent with the previous ones.

A time dependent velocity for the substrate was then introduced in the numerical model as:

(9) $$V\left(t\right)=\bar{V}\left(1+\frac{A_{v}}{\bar{V}}\right)\sin\left(2\pi Ft\right),$$

where *t* is time, *F* is the main frequency of the printing head velocity fluctuations, and $A_{v}$ is its relative amplitude. This evolution is represented by the black line in Figure 20a.

**Figure 23:**
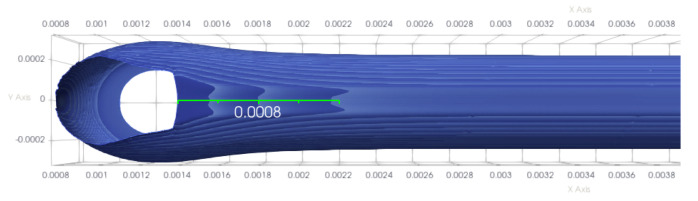
Interface of the strand obtained with a sinusoidal substrate velocity. The ruler has a length of 0.8 mm.

The resulting interface of the strand is shown in Figure 23. It can be seen that the thickness fluctuations are quickly damped by viscous diffusion: they survive only over a distance of approximately 0.8 mm, with the first wavelength of a bit smaller than 0.2 mm. It is interesting to note that the characteristic time of the extrusion flow $t_{extr}=d/U=0.034$ s is of the same order of magnitude than the period 1/F=0.013 s of the fluctuations. It is thus by essence a highly unsteady flow. The fact that we observe the instability experimentally is the proof that glass transition and solidification take place shortly after extrusion, thus “freezing” the thickness fluctuations.

## 4. Conclusions

This paper presents an experimental and numerical study of the extrusion and deposition of a single strand of PLA by FDM. The shape of the meniscus is captured by high-speed visualization of the bead region, and the samples’ morphology is analyzed post solidification. The polymer flow is computed via a 3D finite volume model that assumes purely viscous and isothermal flow, and the predictions are confronted to the experimental data. The main conclusions reached are the following:

- The measurement of the print speed and the deposited volumetric flux of material allows for a precise estimation of the extruded flow velocity.
- The predicted shape of the extruded flow in the meniscus region is well validated by high-speed visualization in the range of parameters studied.
- The average strand shape and dimensions predicted by CFD coincides in general very well with the ones of the solidified samples measured by SEM. This extends the conclusions in Reference [24] to high printing speeds.
- The sinusoidal behavior of the printing head velocity leads to a visible roughness pattern on the strand. This instability is not predicted by the CFD approach used here, presumably because solidification is not accounted for.

Even if it does not consider viscoelasticity nor thermal effects, the CFD model provides reliable predictions for the mean flow of polymer within the range of printing parameters investigated here. Nevertheless, care should be taken in the use of this simplified approach for more extended process windows, in which the extrusion speed, material properties and substrate temperatures are substantially different. Assessing the processability window of any newly formulated material in FDM, with known rheological properties, requires the previous quantification of the importance of a wide variety of physical phenomena.In particular, we find here that further investigation of the strand surface instabilities will require the inclusion of heat transfer and phase change in the numerical model. On the other hand, the deposition of multiple strands adjacent to each other is the natural continuation of this research in order to identify and quantify the different types of defects in FDM printing. Because of filament cooling, reheating, and deformation, the conclusions reached for a single strand on a flat smooth substrate might not be valid when a strand is deposited on a layer of the same material. The simulation of this process is an important step towards the study of the bonding between adjacent filaments, in which strength is paramount to avoid delamination of the printed piece.

## Figures and Tables

**Table 1:** Experimental tests conditions.

Process Parameter	Symbol	Unit	Values
Nozzle separation	*h*	mm	0.2, 0.3
Printing head velocity	*V*	mm/s	15, 22, 30
Extrusion mean velocity	*U*	mm/s	7.5, 11, 15 (h/d=0.5), 11.5, 16.9, 23.1 (h/d=0.75)
Normalized printing head velocity	V/U	-	2 (h/d=0.5), 1.3 (h/d=0.75)
Extrusion temperature	*T*	°C	215

**Table 2:** PLA properties used in the computations.

Property	Symbol	Unit	Value
Density	ρ	kg/m^3^	1250
Dynamic viscosity (newtonian)	η	Pa·s	330
Shear rate dependent viscosity	η	Pa·s	Equation (3)
Surface tension	σ	N/m	0.04
Linear thermal expansion coefficient	$\alpha_{L}$	°C^−1^	$6.8\times 10^{-5}$

**Table 3:** Mesh characteristics.

Mesh	Cell Number	Min. Cell Size
Coarse	600,000	40 μm
Medium	2,400,000	20 μm
Fine	7,700,000	10 μm

## Abbreviations

The following abbreviations are used in this manuscript:

ABS	Acrylonitrile Butadiene Styrene
AM	Additive Manufacturing
CFD	Computational Fluid Dynamics
FDM	Fused Deposition Modeling
FFF	Filament Fused Fabrication
LED	Light Emitting Diode
PET	Polyethylene Terephthalate
PLA	Poly-Lactic Acid
VOF	Volume Of Fluid
